# Evaluating the Electromagnetic Shielding of Continuous Carbon Fiber Parts Produced by Additive Manufacturing

**DOI:** 10.3390/polym15244649

**Published:** 2023-12-08

**Authors:** Luís C. Martins, Cátia S. Silva, Leandro C. Fernandes, Álvaro M. Sampaio, António J. Pontes

**Affiliations:** 1 Institute of Polymers and Composites, Department of Polymer Engineering, Campus de Azurém, University of Minho, 4800-058 Guimarães, Portugal; id6045@alunos.uminho.pt (L.C.M.); catia.silva@dep.uminho.pt (C.S.S.); leandrofernandes@dep.uminho.pt (L.C.F.); 2 DONE Lab—Advanced Manufacturing of Polymers and Tools, Campus de Azurém, University of Minho, 4800-058 Guimarães, Portugal; amsampaio@eaad.uminho.pt; 3 Lab2PT—Landscapes, Heritage and Territory Laboratory, School of Architecture, Campus de Azurém, University of Minho, 4800-058 Guimarães, Portugal

**Keywords:** additive manufacturing, material extrusion, continuous fiber reinforcement, electromagnetic shielding effectiveness, thermoplastic composite, continuous carbon fiber, multi-material

## Abstract

Electronic devices are sensitive to electromagnetic (EM) emissions, and require electromagnetic shielding protection to ensure good operation, and prevent noise, malfunctioning, or even burning. To ensure protection, it is important to develop suitable material and design solutions for electronic enclosures. Most common enclosures are made with metal alloys using traditional manufacturing methods. However, using thermoplastic composites combined with additive manufacturing (AM) technologies emerges as an alternative that enables the fabrication of complex parts that are lightweight, consolidated, and oxidation- and corrosion-resistant. In this research, an AM technique based on material extrusion was used to print 2 mm-thick specimens with a multi-material made of micro-carbon fiber (CF)-filled polyamide that was reinforced at specific layers using continuous carbon fibers stacked with a 90° rotation to each other. The specimens’ electromagnetic shielding effectiveness (EMSE) was evaluated in the frequency band of 0.03–3 GHz using the coaxial transmission line method. Depending on the number of CF layers, the EM shielding obtained can be up to 70 dB, with a specific shielding up to 60 dB.cm^3^/g, predominantly by the absorption mechanism, being 22 times higher than without the CF layers. These findings promote this innovative approach to lightweight customizable solutions for EM shielding applications.

## 1. Introduction

Nowadays, there are many electronic devices used for various applications using a variety of frequency bands, such as wireless LAN (2–6 GHz), Bluetooth (2.4 GHz), mobile phones (0.8–3 GHz), and military communication/radar (8–12 GHz) [1]. These devices, when in operation, emit electromagnetic (EM) fields and are susceptible to electromagnetic interference (EMI) that originates from surrounding devices or from natural sources. Therefore, all electronics must be enclosed by an electromagnetic-impermeable material (shield) that provides mechanical support and prevents signal interference or electrostatic discharge (ESD), which can lead to noise, malfunction, or even burning [2,3]. The efficiency of a barrier, also known as EMI shielding effectiveness (SE), is an EM field ratio between the source and the receptor that can quantify the attenuation of the wave’s propagation through the material or apertures of an enclosure, and is achieved through three different loss mechanisms, namely, reflection, absorption, and multiple reflections [2,3,4,5,6]. SE is expressed in decibels (dBs), and the common requirement for commercial electronics is the range of 40 to 60 dB [7]; however, an SE of 30 dB (99.9% attenuation) is also considered an adequate level of shielding for many applications [8,9].

SE is directly proportional to the material’s electrical conductivity, and that is the reason why conducting metal alloys, such as aluminum, copper, steel, or silver, are broadly used as reflecting EMI shielding materials. However, the high density, poor mechanical flexibility, low resistance to chemicals, low resistance to oxidation, and high reflectance constrict the use of these metallic alloys. Because of these reasons, metallic shields are being replaced by flexible hybrid shields made of metamaterials [10,11], intrinsically conducting polymers [12,13,14], or thermoplastic composites [9,15,16,17].

The use of thermoplastics composites reinforced with carbon allotropes, such as carbon nanotubes (CNT), carbon black (CB), carbon fibers (CF), or graphene (Gr), is very appealing for EMI shielding [13,18,19]. The low density, easy processability, and resistance to corrosion and oxidation of these materials allow many improvements over traditional conductive materials, as more complex, flexible, and lightweight enclosures can be fabricated as a sole part that reduces or eliminates seams, preventing EM radiation leakage and SE dropping [4,6,20]. More recently, the application of MXene-based composites has exhibited promising results in the field of EMI shielding materials. Some studies found that a lower volume of these 2D transition metal carbides and nitrides can produce low-density materials with excellent shielding and eco-friendly properties (above 60 dB), depending on the composition, thickness, and processing method [21,22]. 

Thermoplastic composites have been intensively explored by conventional fabrication techniques, such as compression molding or injection molding [23,24,25,26,27,28,29,30]. However, recently, additive manufacturing (AM) technologies improved significantly, and are becoming more adopted in manufacturing final products. AM is a competitive digital manufacturing process that allows the fabrication of complex and functional geometries, due to the inherent design freedom that the layer-by-layer process enables. In combination with design exploration methods, such as generative design and topology optimization, AM can overcome traditional manufacturing limits and achieve a more efficient product performance while improving manufacturability, by reducing lead time, cost, and material consumption [31,32]. AM processes are commonly divided according to seven categories, namely (i) binder jetting; (ii) direct energy deposition; (iii) material extrusion; (iv) VAT polymerization; (v) material jetting; (vi) sheet lamination; and (vii) laminated object manufacturing (LOM) [33]. Material extrusion (ME) technology consists of a bottom-up process based on the extrusion of material in the filament form onto a building platform in a layer-by-layer process, where the filament is deposited on top of the subsequently deposited layer until the part to be produced is complete. At the end of the deposition, the filament solidifies [34]. The main benefits of the ME process include the ease and relative speed of producing functional products at a competitive cost, and also the large range of materials commercially available, as well as the possibility of developing a customizable material adjusted to the product requirements [34,35]. Regarding the part quality and mechanical properties, these are dependent on process parameters, such as build orientation, layer thickness, layer adhesion, type of infill, air gap, raster angle, and raster width [34,36].

AM, specifically the ME technology, has been used in the development of plastic composite parts with electrical conductivity properties and EM shielding characteristics. Most of the studies report the optimization of the printing process and the manipulation of filament properties by adding conductive fillers in order to improve the required properties, which are either mechanical, electrical, thermal, or electromagnetic [28,37,38,39,40,41,42,43,44]. For example, the addition of CNT as a conductive nano-filler to the polymer filament, or a hybrid combination with one additional filler, such as CB, was developed to improve the electrical conductivity and/or electromagnetic shielding properties of the products printed by ME. Dorigato et al. [38] developed a multi-walled carbon nanotube (MWCNT)-filled acrylonitrile–butadiene–styrene (ABS) compound, showing that the MWCNT improved the tensile, electrical, and thermal properties. Furthermore, they also reported that these properties are also dependent on the printing orientation. Chizari et al. [28] used the ME process to produce conductive microstructures for the functional optimization of lightweight and semi-transparent EMI shields. They formulated a highly conductive carbon nanotubes/polylactic acid (CNT/PLA) printable ink to fabricate 3D scaffolds with significant improvement to the specific EMSE relative to CNT/PLA hot-pressed in solid forms (~70 vs. ~37 dB.cm^3^/g). Schmitz et al. [39,43] fabricated samples via ME with an ABS filled with CNT, CB or a hybrid combination (CNT/CB). They reported that the electrical conductivity, EMSE and mechanical properties of printed parts were considerably dependent on the printing orientation. The EM shielding and respective electrical conductivity values were more efficiently improved with the increase in CNT rather than increasing the CB amount. Furthermore, the EMSE increased with the increased layer thickness, and showed an anisotropic behavior when printed in a perpendicular orientation. Wang et al. [45] produced 3D-printing scaffold structures with a carbon nanotube/polylactic acid composite. The highly conductive CNTs coated on the 3D-printed PLA scaffolds increased the interconnected networks after compression molding, which translated to an enhanced EMI shielding performance as high as 67 dB, while also improving the mechanical robustness of 3D-CNT/PLA. The use of AM methods with graphene-based polymer composites has been indicated as very promising for the enhancement of material properties to enable novel applications in fields such as biomedicine, energy, sensing, and electromagnetic interference shielding [41].

Additive manufacturing can also be used to develop advanced materials as described by Fan et al. [10] and by Lee et al. [44]. By designing complex structures and arranging the distribution of materials with different physical parameters, AM technology provides a direct and efficient way to develop metamaterials with electromagnetic absorption properties [10]. Under the ME printing process of a graphene-polyamide-6 composite filament, it was verified that the introduction of internal geometric assemblies significantly improved EMSE [44]. Moreover, the ME technology was used by Duan et al. [46] to fabricate gradient composite metastructures to effectively absorb microwave signals, proving that the designed metastructure with the thickness of 10 mm can achieve the 10 dB absorbing bandwidth in a frequency range from 5 to 40 GHz.

Recent advances allowed the development of products via a multi-material AM fabrication process of continuous fiber-reinforced polymer composites, with improved performance compared to conventional short fiber-filled filaments. Parmiggiani et al. [47] studied the mechanical resistance of components made with continuous carbon fiber (CCF)-reinforced thermoplastic materials fabricated by ME, with the focus on the influence of the fiber orientation (0°, 45°, and 90°) on the tensile and flexural properties of the produced parts [47]. Blok et al. [48] also used the ME technology from Markforged, Inc. to study the print capability of CCF in order to understand the advantages and limitations of this printing process, in comparison to the printing of chopped short CF-filled polyamide filaments. The tensile strength and stiffness of the CCF printed parts were more than one order of magnitude greater than those of the short fiber-reinforced polyamide printed parts.

Recent technological advances in AM highlight the fabrication of low-cost and high-efficiency complex structures with electromagnetic shielding characteristics. However, the authors are not aware of the existence of peer studies that encompass the use of ME technology to print CCF-reinforced materials to develop a functional enclosure for EMI shielding, hence the relevance of sharing the findings of this study with the scientific and industrial community. 

This paper presents a study regarding the evaluation of the electromagnetic shielding performance obtained by specimens manufactured by ME, using continuous fiber reinforcement materials, considering process parameter variations and specimen thickness. 

## 2. Materials and Methods

### 2.1. Materials

The materials used for the production of the specimens were supplied by Markforged, Inc. (Waltham, MA, USA). The polymeric filament consisted of a chopped micro-CF-reinforced Nylon composite, with the trade name Onyx™. The main properties, provided by the manufacturer, include a tensile modulus of 2.4 GPa, a tensile strain at break of 25%, a flexural strength of 71 MPa, a flexural modulus of 3 GPa, a heat deflection temperature of 145 °C and a density of 1.2 g/cm^3^. Regarding the reinforcement material, a continuous CF filament was selected that presented a tensile modulus of 60 GPa, a tensile strain and break of 1.5%, a flexural strength of 540 MPa, a flexural modulus of 51 GPa, a heat deflection temperature of 105 °C and a density of 1.4 g/cm^3^ [49].

### 2.2. Production

**Figure 1:**
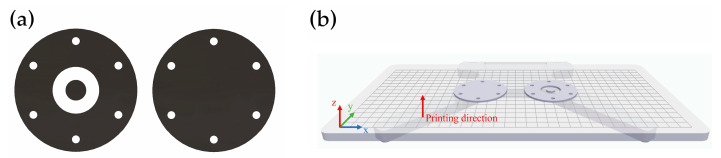
(**a**) CAD representation of the reference specimens; (**b**) build platform depicting specimens positioning for manufacturing, image from software Eiger™ 3D Printing Software version 3.17.21. from Markforged, Inc. (Waltham, MA, USA).

A material extrusion (ME) technology was used to produce two types of specimens, as shown in Figure 1a. The load specimen consists of a flat solid disk with six peripheral holes for fixation on the apparatus that was used for EMSE evaluation, while the reference specimen has a toroid-shaped section removed near the center of the specimen that was used to create a baseline for the EMSE analysis. Both flat disk specimens were built of the same material and have the same diameter of 60 mm and thickness of 2 mm. The specimens were produced with continuous fiber reinforcement (CFR) using the equipment Mark Two™ from Markforged, Inc. (Waltham, MA, USA). Both specimens were built with the positioning shown in Figure 1b.

**Figure 2:**
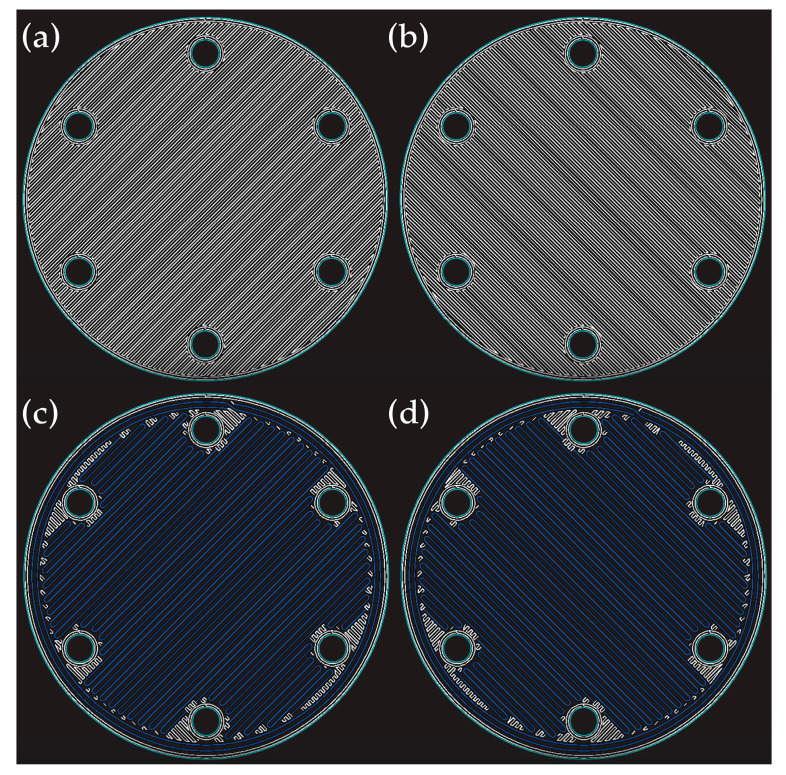
Printing patterns, established by the Eiger™ 3D Printing Software version 3.17.21, for Onyx™ (white) and carbon fiber (blue): Onyx™ with (**a**) −45° pattern and (**b**) 45° pattern, and CF with (**c**) −45° pattern and (**d**) 45° pattern.

For the printing process, a layer height of 0.125 mm and a solid fill pattern (fill density of 100%) were defined for the entire part. The 2 mm-thick specimens were printed using two Onyx™ (O) peripheral wall layers, each 0.8 mm thick. The software option “isotropic fiber” was chosen as the “fiber fill type” to print unidirectional fibers with an alignment angle of either −45° or 45° (Figure 2). The specified alignment angle alternates between layers, up to a total number of 16 layers. This customization was carried out according to the specifications enabled by the Markforged, Inc. cloud software, Eiger™ version 3.17.21.

In this research, a total of 9 types of specimens were printed. One specimen was printed totally with Onyx™, while the remaining specimens were reinforced with continuous carbon fibers in some of the sliced layers of the sample. Since the Eiger™ 3D Printing Software version 3.17.21 locks the first (layer 1) and last layer (layer 16) to be printed with Onyx™, the other 14 layers were used for CF insertion. As previously stated, the CF and Onyx™ were printed in a unidirectional pattern with an alignment angle of either −45° or 45°, which was alternated in each layer. This process was used to produce composite samples with just 1 CF layer up to 14 layers, as shown in Table 1 and Table 2. 

**Figure 3:**
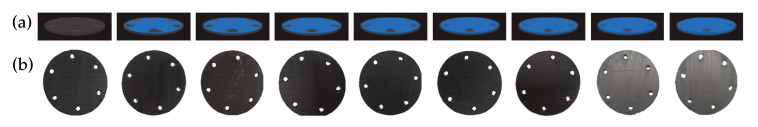
(**a**) Eiger™ model and (**b**) photographic registry of each specimen from left to right: 0 CF, 1 CF, 2 CF, 4 CF, 6 CF, 8 CF, 10 CF, 12 CF and 14 CF, respectively.

Some theoretical characteristics of the specimens were provided by Markforged Eiger™ 3D Printing Software version 3.17.21 and are exhibited in Table 2 and Figure 3. In Figure 3 it is possible to verify the CF volumes (in blue) inside the specimen’s preview model. The Onyx™ baseline specimen (corresponding to 0 CF) highlights the absence of CF as there are no blue outlines. In Table 2, the “print time”, “Onyx™ volume”, “CF volume” and “part mass” characteristics are theoretical estimations provided by the software, while the “part density” is an arithmetic division between the “part mass” and the sum of the constituent’s volumes. Additionally, Figure 3 shows a photo of each produced specimen. Since the base and top layers were both printed in Onyx™, the appearances of the samples are identical.

### 2.3. Characterization

This section presents the characterization procedure defined for the specimen’s production, which includes the assessment of the quality of the specimens and an electrical evaluation based on the electromagnetic shielding effectiveness and electrical conductivity.

#### 2.3.1. Thickness, Weight and Density

Specimen density was measured following Archimedes’ principle. According to this principle, the volume of an immersed body is equal to the volume of the displaced volume. Therefore, a body immersed in a liquid is subjected to a buoyancy force equal to the weight of the liquid displaced by the volume of the body. The specific density is calculated using the equation:

(1) $$\rho =\frac{W_{body,air}\times \rho_{liquid}}{W_{body,air}-W_{body,liquid}},$$

where $W_{body,air}$ is the weight of the body in air, $W_{body,liquid}$ is the weight of the body in the liquid and $\rho_{liquid}$ is the specific density of the liquid.

The procedure for the density measurement was performed with an analytical balance AS 202.R2 from Radwag with SDK 01 density kit from Scaltec Lda (Santarém, Portugal). 

Regarding the measurement of the specimens’ thickness, a Mitutoyo Digimatic Caliper (Neuss, Germany) was used.

#### 2.3.2. Morphology

The morphology of the printed specimens was observed along the thickness cross-section with a Leica DMS1000 digital microscope (Wetzlar, Germany) using a magnification of 6 times.

#### 2.3.3. Electromagnetic Shielding Effectiveness

**Figure 4:**
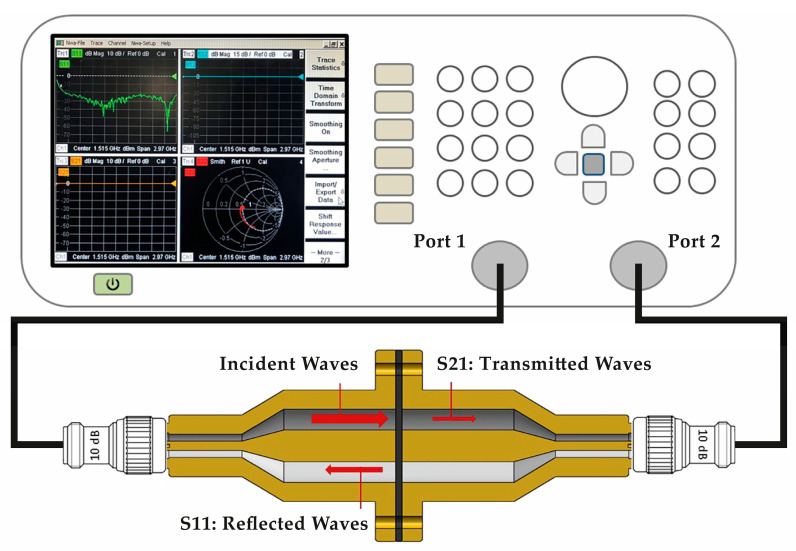
Shielding apparatus schematic representation (the readings displayed on this schematic representation are provided for informational purposes regarding the type of visualization typically presented on a VNA).

EMSE measurements were performed with a test procedure adapted from the ASTM D4935-99 Standard (Standard Test Method for Measuring the Electromagnetic Shielding Effectiveness of Planar Materials) [50], wherein the sample was placed between two coaxial flanges acting both as a sample holder and transverse electromagnetic (TEM) waveguide, as used by Hong et al. [51], Sarto and Tamburrano [52] and Vasquez et al. [53]. The sample holder comprises an enlarged coaxial transmission line, made of a brass alloy designed to support 60 mm-diameter samples maintaining a characteristic impedance of 50 Ω throughout the entire length of the holder, and it is connected to a Vector Network Analyzer (VNA) (R&S^®^ZVL3) from Rohde & Schwarz (Munique, Germany) with the assistance of two coaxial cables and two 10 dB 50 Ω attenuators. Figure 4 depicts the testing apparatus.

Shielding effectiveness (SE) was measured at the frequency range between 30 MHz and 3 GHz, which is a radio frequency spectrum common to automotive standards for electromagnetic compatibility (CISPR 25) [54], and is the VNA limit range. The VNA used an input power of 0 dBm, corresponding to 1 mW, to generate EM waves, and recorded the scattering parameters S11 (reflection) and S21 (transmission) to determine the total EMSE of the material under test. According to the ASTM D4935 standard, the material’s SE can be expressed by the ratio between the transmission scattering parameter *S*21 values of a reference sample (*S*21*_ref_*) and a load sample (*S*21*_load_*), as shown in the following equation:

(2) $$SE=20\log_{10}\frac{{S21}_{ref}}{{S21}_{load}}$$

The EM shielding of a given material can also be described according to the following equation [43,44,55,56,57]:

(3) $${SE}_{T}\left(dB\right)={SE}_{R}+{SE}_{A}+{SE}_{M},$$

where *SE_R_* is the shielding by reflection, *SE_A_* is the shielding by absorption, and *SE_M_* is the shielding by multiple reflections. *SE_R_* and *SE_A_* were calculated using the following equations:

(4) $${SE}_{R}\left(dB\right)=-10\log_{10}\left(1-R\right),$$

(5) $${SE}_{A}\left(dB\right)=-10\log_{10}\left(\frac{T}{1-R}\right),$$

wherein the reflected coefficient (*R*) and transmission coefficient (*T*) were directly obtained as:

(6) $$R={\left|S11\right|}^{2},$$

(7) $$T={\left|S21\right|}^{2},$$

Since the microwave multiple internal reflections (*SE_M_*) can be negligible when *SE_T_* is higher than 10 dB [54], the total shielding was calculated as:

(8) $${SE}_{T}\left(dB\right)={SE}_{R}+{SE}_{A},$$

#### 2.3.4. Electrical Conductivity

The electric volume resistivity (*ρ*) of the filaments was measured according to the Ohms law using a four-point probe method using the Keithley 2635B System SourceMeter and Keithley 5809 (Cleveland, OH, USA) clips according to the equation:

(9) $$\rho =\frac{A}{l}R,$$

where *A* is the area of the filament cross-section, *R* is the resistance and *l* is the distance between the clip electrodes, which is equivalent to the thickness of the filament sample.

Considering that, according to Ohm’s law, the volume resistance (*R*) is derived by dividing the applied test voltage (*V*) measured in volts by the subsequent current (*I*) measured in ampere, the equation changes to:

(10) $$\rho (\Omega .cm)=\frac{A\times V}{l\times I}$$

For the printed specimens, the electrical volume resistivity was measured according to the ASTM D257 standard [58] “Standard Methods of Test for Electrical Resistance of Insulation Materials” by using the Keithley 2635B System SourceMeter (Cleveland, OH, USA) and the Keithley Model 8009 (Cleveland, OH, USA) resistivity test fixture. The volume resistivity, in accordance with the ASTM standard D257, was calculated with the following equation:

(11) $$\rho =\frac{A}{t}R,$$

where ρ is the volume resistivity measured in Ω.cm, *A* is the effective area (cm^2^) of the guarded electrode applied for the measurement, *t* is the average thickness of the specimen measured in cm and *R* is the volume resistance in Ω.

The Keithley Model 8009 (Cleveland, OH, USA) resistivity test fixture uses circular electrodes with an effective diameter of the guarded electrode of 5.40 cm. The effective area (*A*) is calculated based on Equation (11):

(12) $$A=\frac{D_{0}^{2}}{4}\pi ,$$

where $D_{0}$ is the effective diameter measured in cm.

By replacing the effective diameter value, the effective area (*A*) is obtained:

(13) $$A=\frac{{\left(5.40\right)}^{2}}{4}\pi =22.9\;{cm}^{2}$$

Replacing the calculated effective area value (*A*) and considering the Ohms law previously mentioned that enables us to replace *R* with *V/I*, Equation (10) changes to:

(14) $$\rho \left(\Omega .cm\right)=\frac{22.9\times V}{t\times I}$$

The electrical volume conductivity (σ) is the reciprocal of electrical resistivity. Hence, for both the filament and the printed specimens, it can be calculated according to:

(15) $$\sigma \left(S/m\right)=\frac{1}{\rho }$$

## 3. Results and Discussion

This section presents the results and respective discussion, and it is divided into two main points. In the first point is presented a discussion of some aspects related to the quality of the produced specimens, in particular, the measured thickness, weight, and density, in comparison to the estimations provided by the software Eiger™ version 3.17.21. A morphologic evaluation is also shown in relation to the weight and density of the composite specimens. In the second point, the results, and a discussion of the most important aspect of the research, the electromagnetic shielding of the printed composites as a function of the number of continuous CF layers, are presented. Lastly, a brief discussion regarding the performance of these materials in comparison with the materials in the same property category is presented.

### 3.1. Quality of the Printed Composite Specimens

**Figure 5:**
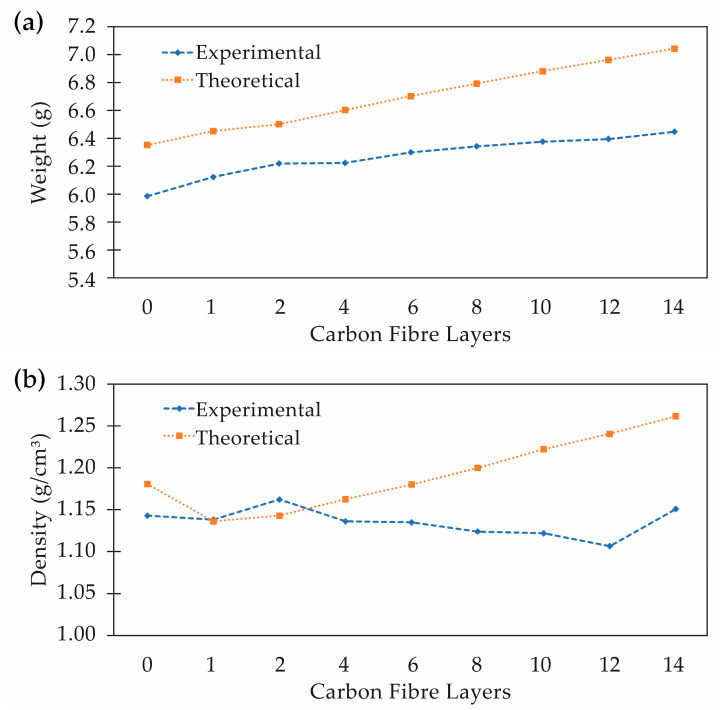
Experimental and theoretical values for (**a**) part weight and (**b**) part density.

The thickness, weight and density were measured for all specimens to assess the differences associated with the different printing conditions. Generally, thickness and density can influence the EM shielding and specific EM shielding, respectively [9,27,59]. Therefore, their evaluation is important in investigating the EM shielding relative to other materials, and is also important for the quality and performance control of the printing process. The respective measured values are shown in Table 3, and a comparative analysis with the theoretical values from the software is shown in Figure 5.

Regarding the specimens’ thickness, the measurements were similar for all samples, near the nominal thickness of 2 mm. Therefore, we conclude that any variation in the shielding performance is derived from the composite content (i.e., Onyx™ and/or CF layers) and the internal morphology along the part thickness.

**Figure 6:**
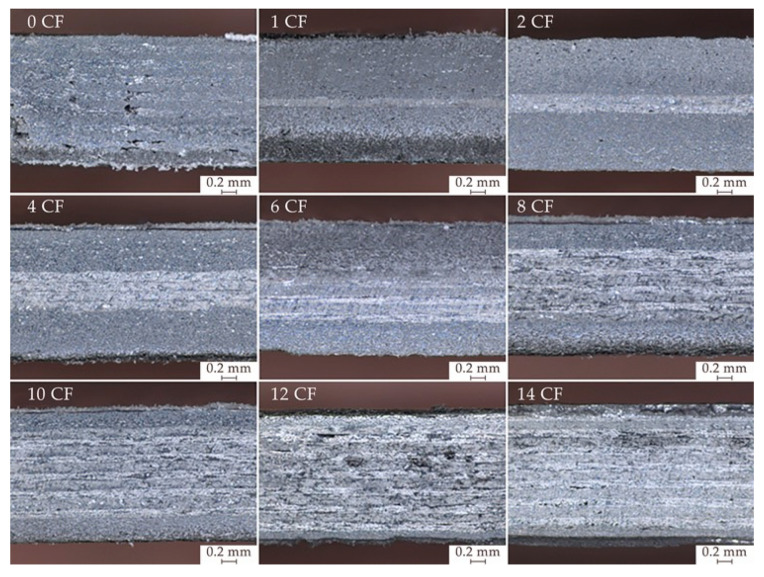
Microscopy of specimens’ cross-section.

As for the weights of the specimens, it can be noticed (as shown in Figure 5a) that the measured values are all bellow the expected values indicated by the software, and near 6% on average. The lowest weight of the specimens is a result of lower real density. The experimental tests indicate that the actual density values are lower than those estimated by approximately 4.6% on average. This variation is due to the porosity inside the specimen; in particular, voids between layers were verified by microscopic analysis, as shown in Figure 6. The existence of porosity leads to gaps between successive layers, impacting the connectivity of the layers and consequently affecting the electrical conductivity and electromagnetic shielding of the specimens. This effect was also observed in the research by Blok (2018) [48]. Furthermore, the density difference compared to nominal values is higher for the specimens with more CF layers, especially for the specimens with 8, 10 and 12 layers of CF (8 CF, 10 CF and 12 CF) where the presence of voids is more evident.

### 3.2. Electromagnetic Shielding

The results and discussion of the electromagnetic shielding evaluation are presented in this section. Considering that sample density can impact the electromagnetic shielding [28,59], a normalization was conducted to account for different density values, such as specific SE, which is detailed in the following discussion. 

**Figure 7:**
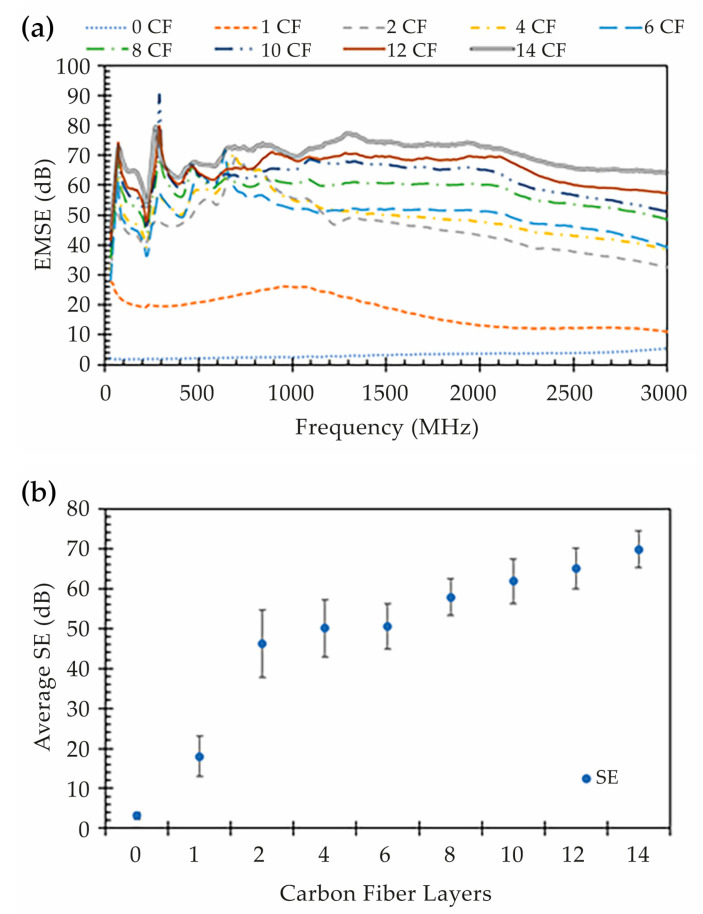
EMSE results: (**a**) EMSE for all specimens as a function of frequency and (**b**) average EMSE as a function of the number of CF layers.

Focusing on the EM shielding analysis, it is possible to observe in Figure 7, and determine using Equation (2), a high improvement in the shielding performance with the printing of continuous CF layers on the internal layers of the composite specimen, from less than 10 dB (0 CF) up to 70 dB (14 CF). This enhancement is greater when a combination of at least two CF layers is used, as noted by the SE jump from one CF layer (1 CF) to two CF layers (2 CF). The two layers tied together create a thicker overlap in the CF printed pattern, which reduces the voids between the deposited CF filaments in the same layer.

With more than 1 CF layer (2 CF up to 14 CF), it is possible to verify that the EMSE shows linear proportionality with the increase in two combined CF layers. This behavior is highlighted in Figure 7b, where we can see that the coefficient of linearity varies with the frequency. However, on average, the EMSE increases by approximately 4 dB with the increase in combinations of CF layers.

Further, when looking at the frequency-variable EMSE results shown in Figure 7a, it is possible to observe that, as expected, the electromagnetic shielding of Onyx™ without CF (0 CF) (line with blue dots) increases with the wave frequency. The shielding of Onyx™ occurs because the filament is filled with chopped CF, which provides some shielding ability to the specimen. However, it remains below 10 dB at the complete frequency range. Furthermore, the data collected from the composite specimens with at least one CF layer show some resonant characteristics below 800 MHz, in contrast to the stable and linear growth seen with Onyx™ (0 CF). This effect is still not fully understood, but it may be due to an antenna effect induced by the continuous CF length.

Additionally, the interaction of the EM wave with specimens with continuous CF layers has a particular effect. It can be noted that, above 1 GHz, the shielding provided by the CF layers decreased with the frequency, as opposed to the effect in specimens produced entirely from Onyx™. The shielding drop can be reasonably explained by the shorter waves traveling through gaps in the mesh screen created by the stacked CF layers, as can be seen in metallic wire meshes, ventilation panels or scaffolds, where the shielding performance is governed by the cross-section and depth of the apertures [3,15,28]. However, this effect was not expected for the wavelengths at which this study was performed, as the dimensions of these gaps or voids were much smaller than half the wavelength.

**Figure 8:**
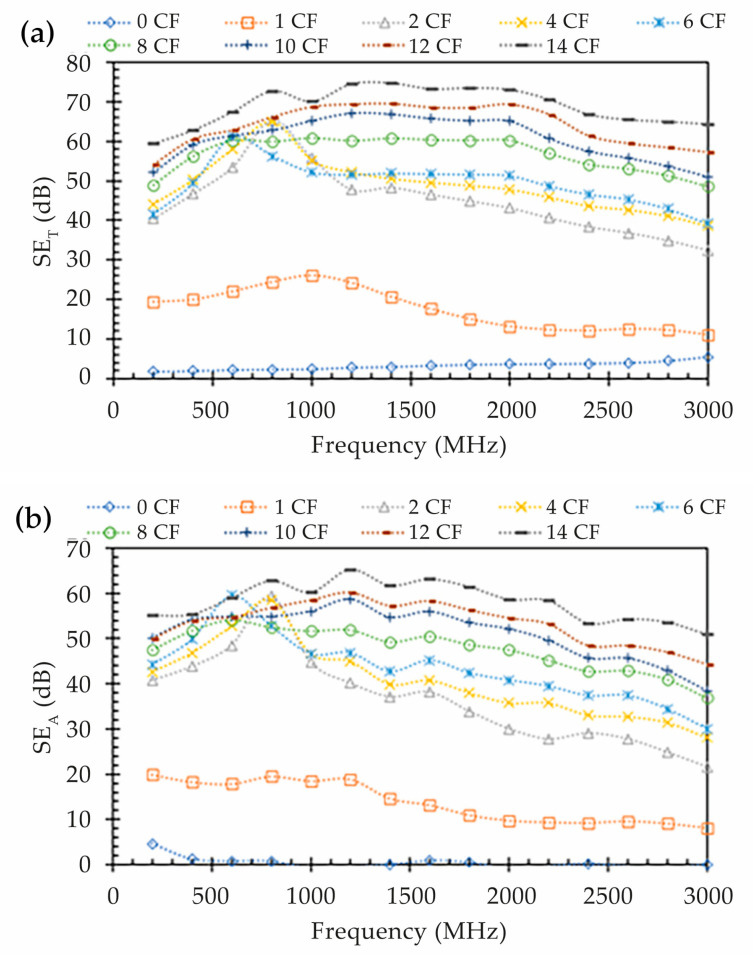

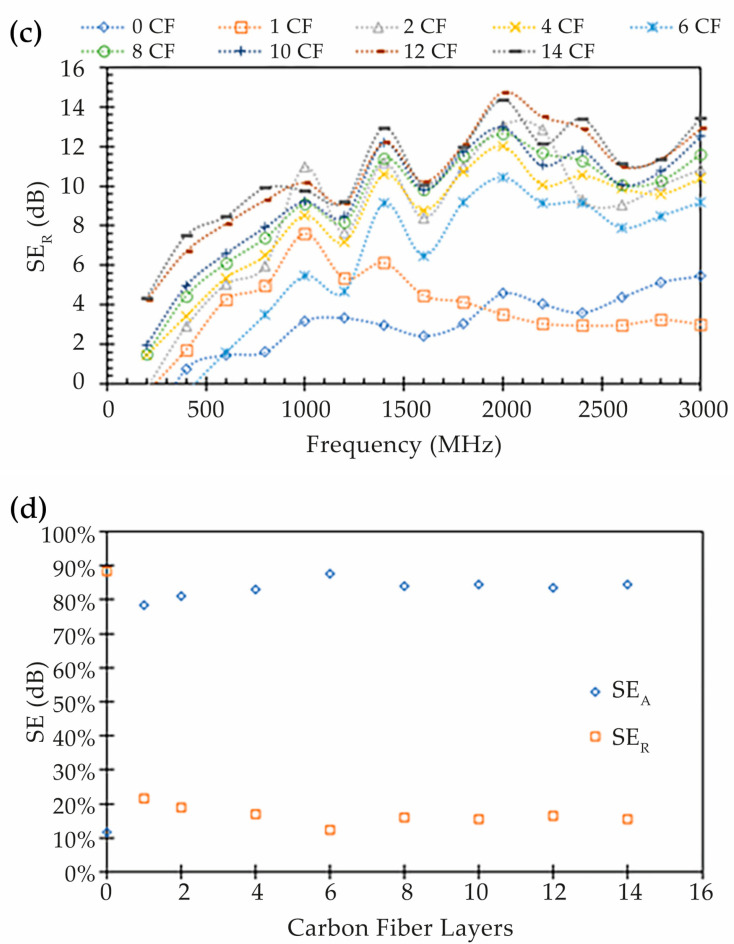
EMSE results: (**a**) total shielding (*SE_T_*) along the frequency range; (**b**) absorption shielding (*SE_A_*) along the frequency range; (**c**) reflection shielding (*SE_R_*) along the frequency range; and (**d**) average ratio for each shielding mechanism.

When analyzing the shielding properties for a given material, it is important to distinguish the discrete mechanisms of absorption (*SE_A_*) (Figure 8b), reflection (*SE_R_*) (Figure 8c), and the total shielding (*SE_T_*), which are a product of the sum of the two components, as described in Equations (4)–(8) from Section 2.

This analysis shows that, except for the Onyx™ specimen (0 CF), absorption is the dominant shielding mechanism for the printed composite specimens. The specimen with 0 CF layers demonstrates negligible shielding by absorption and an increase in reflection shielding with frequency, up to 5.5 dB. For the specimens composed of continuous CF, the absorption shielding increases with the number of CF layers, like the total EM shielding previously discussed. Furthermore, the absorption effect appears to decrease at higher frequencies. In the case of the composite made with 2 CF layers, the measured absorption shielding is above 40 dB at lower frequencies and decreases to approximately 20 dB at 3 GHz, while for the specimens composed of 14 CF layers, the shielding by absorption is above 55 dB, with a peak value of 65 dB at 1.2 GHz, and it decays to near 50 dB at the upper frequency of 3 GHz. In contrast, the shielding by reflection observed in the printed composites with more than one CF layer appears to increase with frequency, but shows less relevance to the overall shielding, with observed values lower than 15 dB. The absorption of EM waves in the specimens printed with continuous CF is responsible for approximately 80% of the shielding behavior. Hence, the printing of continuous CF layers can result in rather suitable radar absorber materials.

The main reason for the shielding improvement with the addition of more CF layers is the increase in relative thickness of a material, in this case continuous CF, which has much higher electrical conductivity than the chopped CF inside the Onyx™ baseline material. This interpretation can be corroborated by the electrical conductivity measured for both Onyx™ and CF filaments before they underwent the printing process, as shown in Table 4. The electrical conductivity results demonstrate that the continuous CF has a conductivity between 13 to 143 S/m (electrical resistivity between 1 to 23 Ω.cm), which is almost 10 orders of magnitude higher than the electrical conductivity of Onyx™. Hence, since shielding is proportional to the material electrical conductivity and thickness, it is expected that the existence of a larger layer of the conductive continuous CF will lead to increased shielding effectiveness. However, since the printing of continuous CF is restricted to the inner layers of the specimen (Onyx™ printed on the bottom and top layers), the improvement of electrical volume conductivity (reduction in electrical resistivity) with the increase in CF layers was not observed in the experimental results. All composite specimens exhibited an electrical conductivity in the order of 1 × 10^−10^ S/m (electrical resistivity in the order of 1 × 10^11^ Ω.cm), which is near the values measured for the specimen without continuous CF.

**Figure 9:**
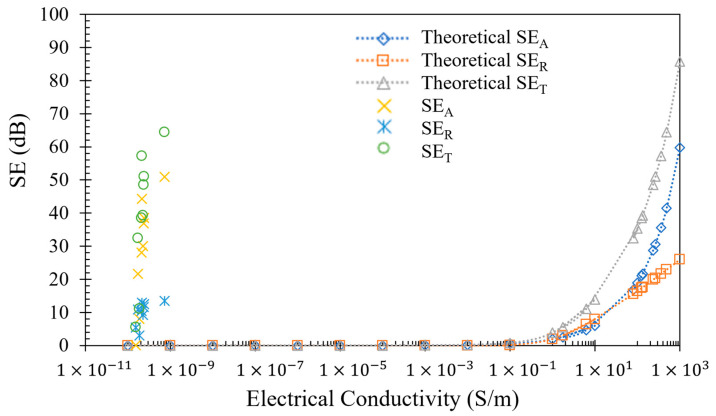
EM shielding and electrical volume conductivity relation. EMSE is expected to increase for higher conductivities with a power law.

When attempting to deduce the EM shielding effectiveness based on the electrical conductivity measured in the as-built specimens, it is possible to observe that, for this specific type of material, the shielding estimations are likely to be underestimated and fail to represent the actual measured values illustrated in the previous figures. As depicted in Figure 9, an electrical conductivity in the order of 10^−10^ S/m (electrical volume resistivity exceeding 10^11^ Ω.cm) would suggest almost negligible shielding. However, the real shielding of the specimens ranges from 10 dB to 70 dB, depending on the number of carbon fiber (CF) layers. To achieve this level of shielding requires electrical conductivity near to or above 5 S/m (electrical resistivity near to or below 20 Ω.cm), which aligns with the conductivity measured for the isolated carbon fibers. Thus, the intrinsic electrical properties of the carbon fibers should be considered when calculating EM shielding using theoretical models.

**Figure 10:**
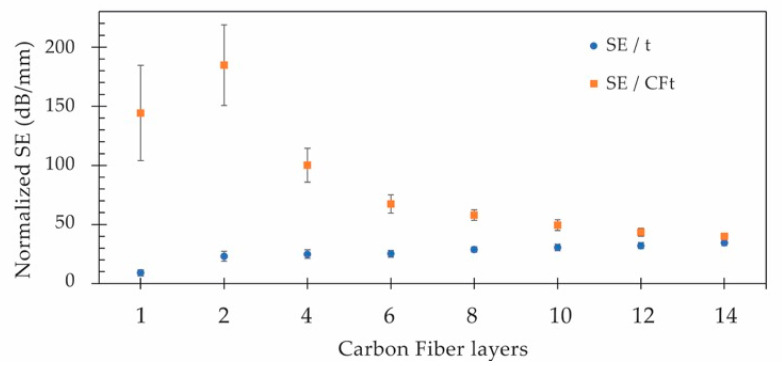
Normalized shielding effectiveness for the specimen’s thickness (t) and per added CF layer thickness (CFt).

**Figure 11:**
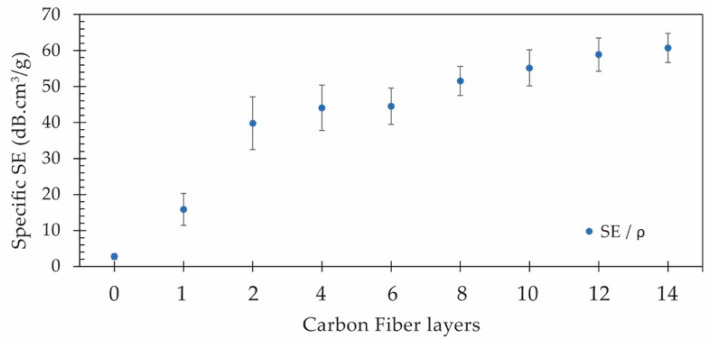
Specific EM shielding for each composite formulation.

As previously mentioned, and reported in other studies [28,44,59], it is important to investigate the impact of the composite thickness and density on the overall electromagnetic shielding in order to assess the shielding performance in relation to different materials. Consequently, the normalized shielding (SE divided by the specimen’s thickness) and the specific shielding (SE divided by the specimen’s density) were determined and are displayed in Figure 10 and Figure 11, respectively.

As evident from the data depicted in the blue circles, the developed composites, featuring a minimum of two CF layers, exhibit a normalized shielding (SE/t) ranging from 23 dB/mm to 34 dB/mm, contingent upon the number of CF layers. This linear growth, escalating by a factor of 1.2 with each additional pair of CF layers, would typically suggest the potential to enhance shielding by approximately 30 dB for each additional millimeter of thickness if the composites were homogeneous. However, the shielding effect primarily arises from the inner CF layers within the overall composite thickness. Hence, one can tailor the normalized shielding by factoring in the isolated CF layer thickness through the division of the measured EMSE by the effective CF thickness (SE/CFt). The adjusted values (depicted as orange squares) show that the CF layer can achieve an SE of nearly 185 dB/mm for the composite with two CF layers, with the shielding effectiveness diminishing as the combined CF layers increase, following a power-law function with an average power of −0.7. 

In terms of specific shielding (SE/*ρ*), this metric assumes significance when manufacturing lightweight components. A higher specific shielding implies the potential to achieve superior electromagnetic barriers with lightweight materials, a crucial aspect for energy conservation. When observing Figure 11, it becomes apparent that beyond two CF layers, these composites exhibit specific shielding abilities ranging from approximately 40 dB.cm^3^/g to 60 dB.cm^3^/g.

In comparison to other composites manufactured through additive manufacturing, particularly ME technology, it is evident that the printed composites utilizing continuous carbon fiber demonstrate superior performance in contrast to materials produced by other researchers. The printed composites, featuring a minimum of two CF layers, exhibit an average shielding ranging from 45 to 70 dB, a normalized shielding ranging from 23 to 34 dB/mm, and a specific shielding ranging from 40 to 60 dB.cm^3^/g. This performance notably surpasses the average shielding of 30 dB, the normalized shielding of approximately 21 dB/mm, and the specific shielding of about 42 dB.cm^3^/g achieved in related studies [28,39,43,44,45]. Notably, given the novelty of employing the additive manufacturing in continuous CF for electromagnetic shielding applications, a direct comparison with peer studies is not possible. Nonetheless, a comparison with materials generated through conventional molding technologies, such as injection molding [15,28] or compression molding [54,56,60,61,62], reveals that the developed material achieves comparable or superior performance to these materials.

## 4. Conclusions

The utilization of the material extrusion technique for continuous carbon fiber printing facilitated the production of composite parts with electromagnetic shielding, capable of achieving up to 70 dB in the frequency range of 0.03–3 GHz, marking an enhancement of more than 22 times compared to the baseline Onyx™-printed composite polyamide material without a continuous carbon fiber layer. From a commercial perspective, materials achieving electromagnetic shielding above 30 dB, blocking over 99.9% of electromagnetic waves, are deemed suitable for practical applications. Therefore, this research demonstrates an innovative and customizable approach to developing lightweight enclosures designed for electromagnetic shielding purposes. 

One notable benefit of these composites lies in their adaptability to performance requirements via modifications of the internal structure. Depending on the carbon fiber layers and the targeted frequency, these composites can achieve electromagnetic shielding efficiency ranging from 40 to 70 dB, with the potential for even higher efficiency in thicker specimens. The impact of the number of carbon fiber layers was evaluated, revealing that the addition of two carbon fiber layers led to a linear increase in shielding at an approximate rate of 4 dB, corresponding to an effectiveness increase of nearly 2 dB/mm. However, beyond two carbon fiber layers, the incremental shielding effectiveness of additional carbon fiber diminishes following a power-law function with a power of −0.7. 

This study further highlights that the additive manufacturing composite materials exhibit absorption shielding (*SE_A_*) ranging from 80% to 90%, indicating an absorption-dominated shielding mechanism. The specific shielding of up to 60 dB.cm^3^/g positions these additive manufacturing composites as a potential, novel lightweight solution for electromagnetic shielding, particularly in applications requiring a high absorption rate.

## Figures and Tables

**Table 1:** Design layout for the produced specimens. O is Onyx™ and CF is carbon fiber.

Layers	Designation of Specimens (O—Onyx™; CF—Carbon Fiber)								
	0 CF	1 CF	2 CF	4 CF	6 CF	8 CF	10 CF	12 CF	14CF
16	O 45°	O 45°	O 45°	O 45°	O 45°	O 45°	O 45°	O 45°	O 45°
15	O −45°	O −45°	O −45°	O −45°	O −45°	O −45°	O −45°	O −45°	CF −45°
14	O 45°	O 45°	O 45°	O 45°	O 45°	O 45°	O 45°	CF 45°	CF 45°
13	O −45°	O −45°	O −45°	O −45°	O −45°	O −45°	CF −45°	CF −45°	CF −45°
12	O 45°	O 45°	O 45°	O 45°	O 45°	CF 45°	CF 45°	CF 45°	CF 45°
11	O −45°	O −45°	O −45°	O −45°	CF −45°	CF −45°	CF −45°	CF −45°	CF −45°
10	O 45°	O 45°	O 45°	CF 45°	CF 45°	CF 45°	CF 45°	CF 45°	CF 45°
9	O −45°	CF −45°	CF −45°	CF −45°	CF −45°	CF −45°	CF −45°	CF −45°	CF −45°
8	O 45°	O 45°	CF 45°	CF 45°	CF 45°	CF 45°	CF 45°	CF 45°	CF 45°
7	O −45°	O −45°	O −45°	CF −45°	CF −45°	CF −45°	CF −45°	CF −45°	CF −45°
6	O 45°	O 45°	O 45°	O 45°	CF 45°	CF 45°	CF 45°	CF 45°	CF 45°
5	O −45°	O −45°	O −45°	O −45°	O −45°	CF −45°	CF −45°	CF −45°	CF −45°
4	O 45°	O 45°	O 45°	O 45°	O 45°	O 45°	CF 45°	CF 45°	CF 45°
3	O −45°	O −45°	O −45°	O −45°	O −45°	O −45°	O −45°	CF −45°	CF −45°
2	O 45°	O 45°	O 45°	O 45°	O 45°	O 45°	O 45°	O 45°	CF 45°
1	O −45°	O −45°	O −45°	O −45°	O −45°	O −45°	O −45°	O −45°	O −45°

**Table 2:** Load specimens’ characteristics.

Layers	Designation of Specimens (O—Onyx™; CF—Carbon Fiber)								
	0 CF	1 CF	2 CF	4 CF	6 CF	8 CF	10 CF	12 CF	14CF
Print time (min)	51	57	60	64	67	75	80	84	85
CF ∑ layer (mm)	0	0.125	0.25	0.5	0.75	1	1.25	1.5	1.75
Onyx™ (cm^3^)	5.39	5.41	5.14	4.57	4.01	3.43	2.85	2.27	1.68
CF volume (cm^3^)	0	0.25	0.56	1.12	1.68	2.24	2.79	3.35	3.91
Part mass (g)	6.36	6.46	6.51	6.61	6.71	6.8	6.89	6.97	7.05
Part density (g/cm^3^)	1.18	1.14	1.14	1.16	1.18	1.20	1.22	1.24	1.26

**Table 3:** Specimens’ average physical dimensions.

ID	0 CF	1 CF	2 CF	4 CF	6 CF	8 CF	10 CF	12 CF	14CF
Thickness (mm)	1.99	2.00	2.01	2.01	2.00	2.02	2.03	2.03	2.03
Weight (g)	5.994	6.131	6.228	6.232	6.308	6.351	6.384	6.403	6.455
Density (g/cm^3^)	1.142	1.137	1.161	1.135	1.134	1.123	1.121	1.106	1.150

**Table 4:** Measured volume electrical conductivity (σ) and resistivity (ρ) for both filaments and printed specimens.

Filament	Onyx™				Carbon Fiber				
	Pre-Processing		Post-Processing		Pre-Processing			Post-Processing	
σ (S/m)	4.88 × 10^−9^		1.38 × 10^−8^		142.89			13.13	
ρ (Ω.cm)	2.11 × 10^10^		8.16 × 10^9^		1.18			23.32	
**Specimen**	**0 CF**	**1CF**	**2 CF**	**4 CF**	**6 CF**	**8 CF**	**10 CF**	**12 CF**	**14 CF**
σ (S/m) × 10^−10^	1.52	1.87	1.71	2.08	2.27	2.36	2.40	2.13	7.30
ρ (Ω.cm) × 10^11^	6.59	5.36	5.86	4.81	4.41	4.24	4.16	4.69	1.37

## Data Availability

Data are contained within the article.
